# The association between anemia and depression in older adults and the role of treating anemia

**DOI:** 10.1002/brb3.2973

**Published:** 2023-03-23

**Authors:** Tamer Ahmed, Catherine Lamoureux‑Lamarche, Djamal Berbiche, Helen‐Maria Vasiliadis

**Affiliations:** ^1^ Faculté de médecine et des sciences de la santé Université de Sherbrooke Sherbrooke Quebec Canada; ^2^ Centre de recherche Charles‐Le‐Moyne (CRCLM) Longueuil Quebec Canada

**Keywords:** anemia, anemia treatment, depression, old age

## Abstract

**Objectives:**

To investigate the association between anemia and depression and whether the treatment of anemia modifies the effect of the association between anemia and depression.

**Methods:**

This secondary data analysis is based on data from the Enquête sur la santé des aînés (ESA)‐Services study conducted in 2011–2013 on community‐dwelling older adults recruited in primary care and have given access to their medico‐administrative data (*n* = 1447). The presence of anemia was self‐reported, as was depression (major and minor) aligned with symptoms of the DSM‐5. Treated anemia was based on the presence of medications delivered to participants. Cross‐sectional associations were analyzed using multivariable logistic regression, controlling for confounders.

**Results:**

The prevalence of self‐reported anemia in our sample was estimated at 6.7%. Self‐reported anemia was associated with increased odds of depression. Individuals with untreated anemia had a 2.6‐fold increased odds of depression compared to those with no anemia. In contrast, the odds of depression in individuals with treated anemia were not different from individuals with no anemia.

**Conclusion:**

The findings underline the importance of treating anemia in older adults. Future longitudinal studies are needed to replicate the findings and further explore the role of treating anemia on symptoms of depression.

## Graphical abstract

Anemia treatment and depression prevalence in old age.

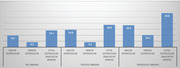

## 1 INTRODUCTION

Anemia is characterized by a lower number of red blood cells or hemoglobin concentration within them leading to a decreased capacity of the blood to meet the body's physiologic needs (Ciesla, 2007). Generally, prevalence estimates of anemia increase with age (Le, 2016) and are higher in low‐middle income countries (Payne et al., 2018). In a systematic review, estimates of anemia prevalence reported in the literature range from 2.9% to 61% in older men and from 3.3% to 41% in older women (Beghé et al., 2004). The variability of these estimates is linked to study settings, the health status of participants, and the criteria used to define anemia.

The most common causes of anemia in old age include nutritional deficiencies, particularly iron deficiency, with folate and vitamin B12 deficiencies as also important causes (Joosten et al., 1992). Anemia of chronic disease is the second most prevalent cause of anemia after iron deficiency anemia (Weiss & Goodnough, 2005). Other well‐known causes of anemia include chronic kidney disease, decreased erythropoietin secretion, reduced response to erythropoietin, inflammatory processes, bone marrow failure conditions, and polypharmacy (Bach et al., 2014; Smith, 2009). A Statistics Canada population survey showed that anemia among seniors was in part due to insufficient mean corpuscular volume and serum ferritin levels, as well as vitamin B12 deficiency but not red blood cell folate (Cooper et al., 2012). Individuals reporting income in the lower quartile were also more likely to report anemia. Anemia is also associated with symptoms such as fatigue, weakness, dizziness, falls, depression, decreased quality of life, hospitalizations, and mortality (Ahmed & Vasiliadis, 2021; Corona et al., 2018; Culleton et al., 2006; den Elzen et al., 2009; Kikuchi et al., 2001).

Depression is a common mental illness worldwide, with an estimated 3.8% of the world's population affected and a prevalence of approximately 5.7% among adults aged 60 years and over (WHO, 2021). Chronic conditions are more prevalent in old age, such as cardiovascular diseases, diabetes, and polypharmacy; all considered risk factors for and complications of depression (Holt et al., 2014; Kilzieh et al., 2008; Minicuci et al., 2002; Spandel et al., 2016). Depression in old age constitutes a significant burden on health care systems with growing demand for mental health services (Liu et al., 2020).

A growing body of literature has investigated and shown a relationship between anemia and depression in older adults (Corona et al., 2014; Onder et al., 2005; Stewart & Hirani, 2012). Others also reported that older adults with anemia and poor cognition at baseline had a five‐fold increased risk of depression after 4 years of follow‐up (Ahmed & Vasiliadis, 2021). However, to date, none of these previously mentioned studies assessed the effect of treating anemia on the prevalence of depression in old age. This study extends the current body of literature by using data on anemia treatment to examine the association between anemia and depression. The objectives are to examine the association between the presence of symptoms of depression (minor and major depression) in older adults and anemia and receipt of treatment and examine whether the treatment of anemia modifies the effect of the association between anemia and depression.

## 2 METHODS

### 2.1 Study population and procedures

The “Étude sur la Santé des Aînés” (ESA)‐Services study consists of a convenience sample of 1811 French‐speaking older adults aged ≥65 years. Participants were recruited in primary care practices between 2011 and 2013 while waiting for medical services in general practice clinics in one of the most populated administrative health regions of the province of Quebec in Canada and representing rural, semiurban, and urban areas. Study recruitment has been described elsewhere in detail (Préville et al., 2014).

Participants visiting one of the participating general practitioners (GPs) received a pamphlet describing the study objectives and an invitation to participate in a face‐to‐face interview with the study coordinator within 30 days. Data were collected through detailed structured computer‐assisted interviews during approximately 90 min at the participant`s home. The ESA‐Services questionnaire included measures on sociodemographic, behavioural, and health information. Written informed consent was obtained from all participants before the interview. Individual data from the ESA‐Services questionnaire were linked to medico‐administrative data from the pharmaceutical registry of the Régie de l'Assurance Maladie du Québec (RAMQ) for the 3 years preceding and following the interview for those who consented. This database provides information on all prescriptions delivered under the provincial public drug plan (drug ID number, type, dosage, duration). Written consent was also obtained to link participants’ health surveys and medico‐administrative data. This secondary study was approved by the ethics committee of the CIUSSS de l'Estrie—CHUS.

**FIGURE 1:**
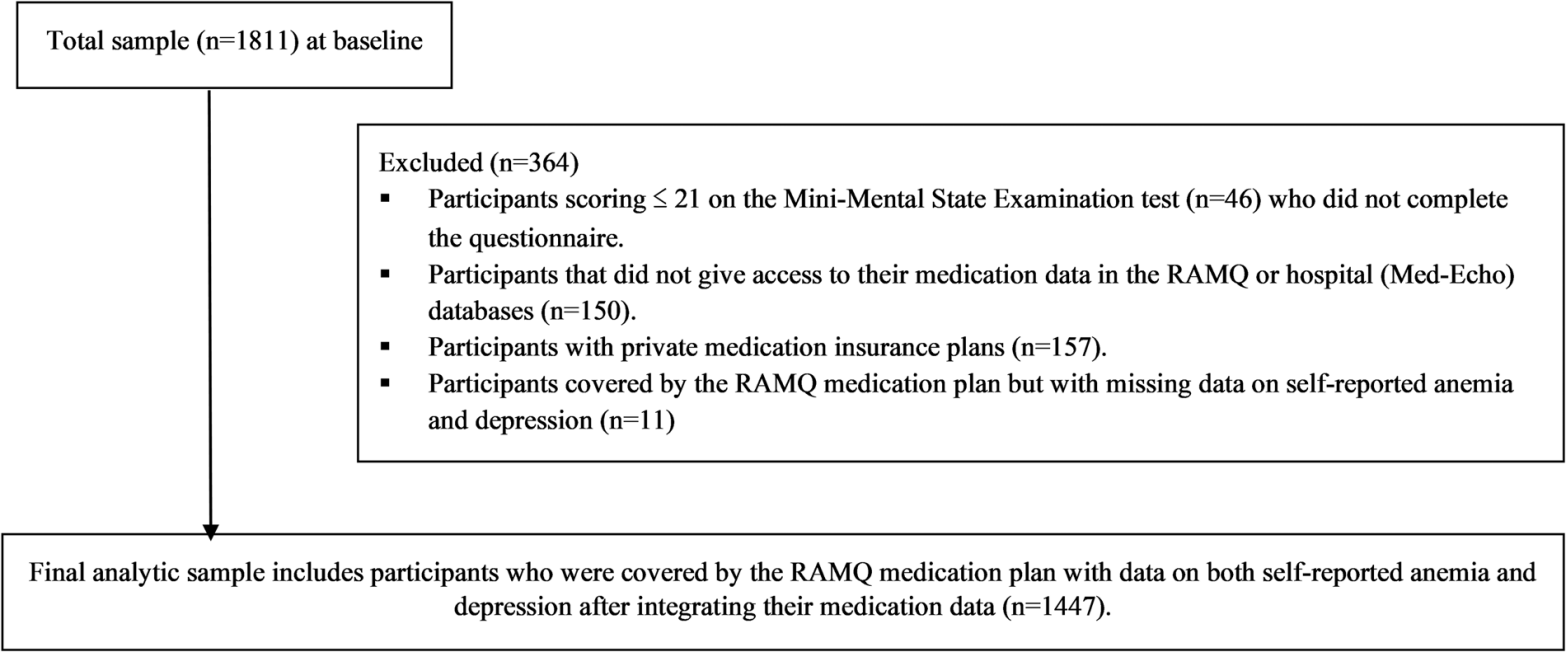
The flow of participants through the ESA‐Services study for the analytic sample.

The current secondary analysis is based on an analytic sample of *n* = 1447 participants for whom complete data on anemia and depression were available and were covered under the RAMQ's public drug plan. The flow of participants through the ESA Services Study is presented in Figure 1.

## 3 MEASURES

### 3.1 Anemia and treatment

Self‐reported anemia was assessed by asking participants if they were currently anemic or if a doctor told them they were currently anemic. In addition, treatment options (generic and brand names) for anemia in Canada were identified (Lim, 2021). Anemia treatment was assessed by the presence of at least one oral or parenteral prescription for anemia treatment (such as oral iron salts, or polysaccharide‐iron complex or heme iron polypeptides or parenteral iron or vitamin B12 or Folic Acid or erythropoiesis‐stimulating agents) in the past 3 years in the pharmaceutical registry of the RAMQ. Participants were then classified into three groups: no self‐reported anemia, self‐reported anemia and treated, and self‐reported anemia but not treated.

### 3.2 Depression

The presence of minor and major depression in the last 6 months was based on symptoms aligned with DSM‐five criteria (Kocsis, 2013) with the exception of criteria regarding the duration of the disorder, social functioning and attribution to a drug or a physical disease. Participants were considered depressed if they had symptoms of major or minor depression. Otherwise, they were classified as not depressed. Participants were classified as having major depression if they showed the essential features of depression (either depressed mood or the loss of interest or pleasure in usual daily activities nearly every day and most of the day for at least two consecutive weeks) and reported at least five of the nine symptoms of depression where at least one of these symptoms is either a depressed mood or loss of interest or pleasure in usual daily activities. Participants were classified as having minor depression if they showed one of the essential features of depression and reported between two and four of the nine symptoms of depression, where at least one of these symptoms is either a depressed mood or loss of interest or pleasure in usual daily activities. Additional analyses also considered the criteria related to the duration (lasting most or all of the day) and frequency (every day or the majority of the days) of symptoms of depression lasting more than two weeks (Kocsis, 2013).

### 3.3 Confounders

The following factors were considered potential confounders based on previous literature (Ahmed & Vasiliadis, 2021): age, sex, marital status, education, income, cigarette smoking, alcohol consumption, body mass index (BMI), self‐reported chronic conditions, and functional status. Participants were classified according to their marital status into married/common‐law partner or separated/divorced/widowed/never married. Education was categorized as primary, secondary, or postsecondary/university. Income was self‐reported and dichotomized as <$25,000 or ≥$25,000. Participants were categorized according to their smoking status into never smokers, ex‐smoker, and current smokers. Alcohol drinking status was categorized as a nondrinker or drinker over the past 6 months. BMI was classified according to World Health Organization categories: underweight (BMI < 18.5), normal weight (BMI 18.5–24.9), overweight (BMI 25–29.9), and obese (BMI > 30) (WHO, 2011). The number of self‐reported chronic diseases was used as a continuous variable. It was calculated by summing the presence of the following health conditions: hypertension, diabetes, lung diseases, cancer, heart diseases, arthritis, and osteoarthritis, liver disease, kidney disease, eye diseases. The presence of functional limitation was based on questions from the revised Système de Mesure de l'Autonomie Fonctionnelle (SMAF)‐IADL subscale (Demers et al., 2010; Desrosiers et al., 1995). Participants provided information on their perception from 1 to 5, ranging from autonomous to dependent, in performing the following eight tasks: housekeeping, preparing meals, shopping, doing the laundry, using the telephone, using means of transportation, taking medications and handling finances. Responses in the current study were scored from 8 to 40, with higher scores indicating higher functional limitations, and categorized into low versus high functional limitations based on quartiles.

### 3.4 Statistical analysis

Descriptive characteristics were reported as mean (standard deviation) or frequency (percentage). Differences in baseline characteristics by self‐reported anemia status (no vs. yes) were assessed using independent *t*‐test for continuous variables, chi‐square tests or Fisher's exact test as appropriate for categorical variables. Logistic regression was used to examine the cross‐sectional associations between self‐reported anemia and depression and the effect of treating anemia on these associations at baseline. Multivariable logistic regression analyses were undertaken using a two‐stage, backward elimination procedure. First, backward elimination (a criterion to keep *p* ≤ .1) was used to improve model fit. Second, variables not significantly contributing to the fit were removed sequentially from the model. Initially, the set of potential confounders was assessed to determine those associated with depression with a *p* ≤ .1. All confounders met this criterion and were included in the subsequent model. Odds ratios (OR) and 95% confidence intervals reported in the table were estimated from the coefficients provided by the final reduced models. All analyses were performed using Stata version 17 (StataCorp, College Station, Texas), and a *p* value of less than .05 was considered statistically significant.

## 4 RESULTS

### 4.1 Characteristics of participants

The characteristics of the study population (*n* = 1447) are shown in Table 1. The mean age of the participants was 73.3 (*SD* = 6.1), with older participants being more likely to self‐report anemia at baseline. Around 57.2 % of participants were women, and the prevalence of self‐reported anemia was 7.1% in men and 6.4% in women. The highest prevalence of self‐reported anemia was observed among participants who reported a higher number of chronic conditions, were nonalcohol drinkers over the past 6 months (11.8%), were underweight (13.6%) or obese (8.1%), reported high functional limitations (11.2%), and depression (10.8%) at baseline (*p* < .05). The prevalence of self‐reported anemia was not statistically different by sex, marital status, education, income, and smoking status (*p* > .05).

**TABLE 1:** Baseline characteristics of the study population by anemia status, according to demographic, lifestyle factors, comorbidities: ESA Study

	Total sample (*n* = 1447)		
	Anemia		
Variable	No (*n* = 1350)	Yes (*n* = 97)	*p* Value*
	*n* (%)/Mean (*SD*)	*n* (%)/Mean (*SD*)	
Age (years)	73.2 (6.1)	74.7 (5.9)	.01
Sex			.61
Men	576 (92.9%)	44 (7.1%)	
Women	774 (93.6%)	53 (6.4%)	
Marital status			.71
Married/common law	851 (93.1%)	63 (6.9%)	
Divorced/separated/ single/never married	499 (93.6%)	34 (6.4%)	
Education			.3
Primary (1–7 years)	339 (92.9%)	26 (7.1%)	
Secondary (8–12 years)	605 (92.5%)	49 (7.5%)	
Postsecondary/ University	406 (94.9%)	22 (5.1%)	
Income			.46
Family income < 25,000 CAD	465 (92.6%)	37 (7.4%)	
Family income ≥ 25,000 CAD	885 (93.7%)	60 (6.3%)	
Cigarette smoking status			.97
Never smoked	511 (93.4%)	36 (6.6%)	
Ex‐smoker	723 (93.3%)	52 (6.7%)	
Current smoker	116 (92.8%)	9 (7.2%)	
Alcohol drinking			<.001
Nondrinker over the past 6 months	335 (88.2%)	45 (11.8%)	
Drinker over the past 6 months	1015 (95.1%)	52 (4.9%)	
BMI			.03
<18.5	19 (86.4%)	3 (13.6%)	
18.5–24.9	412 (92.4%)	34 (7.6%)	
25–29.9	549 (95.6%)	25 (4.4%)	
≥30	338 (91.9%)	30 (8.1%)	
Self‐reported chronic conditions	1.8 (1.3)	2.7 (1.4)	<.001
Functional limitations			<.001
No	944 (94.6%)	54 (5.4%)	
Yes	301 (88.8%)	38 (11.2%)	
Total depression (major or minor)			.02
No	1160 (94.0%)	74 (6.0%)	
Yes	190 (89.2%)	23 (10.8%)	
Major depression			.05
No	1216 (93.8%)	81 (6.2%)	
Yes	134 (89.3%)	16 (10.7%)	
Minor depression			.19
No	1294 (93.5%)	90 (6.5%)	
Yes	56 (88.9%)	7 (11.1%)	

^*^ *p* values indicate the significance of the difference between anemic and nonanemic, using an independent *t*‐test for continuous variables, or chi‐square tests or Fisher's exact test as appropriate for categorical variables.

**FIGURE 2:**
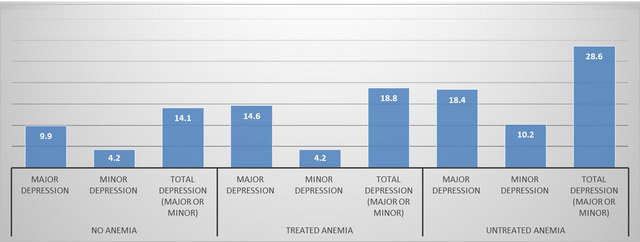
The prevalence of major, minor, and total depression (*n* = 1447) by anemia status and treatment of anemia, ESA services study.

Depression varied significantly by anemia status and whether anemia was treated or not treated (*p* = .02). The highest prevalence of depression was reported among participants with untreated anemia (28.6%), while the lowest prevalence was observed among those who did not self‐report anemia (14.4%). The prevalence of depression among those with treated anemia was 18.8%. A similar trend was observed for major and minor depression by anemia and treatment status (Figure 2).

### 4.2 Cross‐sectional analyses

Multivariable analyses of the association between self‐reported anemia and depression are shown in Table 2. There was no evidence of a multiplicative interaction by sex in the fully adjusted models. Individuals reporting anemia had 82% (*p* = .03) higher odds of depression than those not reporting anemia. Similar estimates were observed for major and minor depression; however, these did not reach statistical significance in the model controlling for potential confounders.

**TABLE 2:** Unadjusted and Multivariable‐adjusted Logistic regression models: associations between anemia and depression, baseline data: The ESA Services Study

	Model 1**^a^**		Model 2	
	OR (95% CI)	*p* Value	OR (95% CI)	*p* Value
**Total depression (reference category: no depression)**				
Anemia vs. no anemia	1.90 (1.16;3.11)	.01	1.82 (1.06;3.13) **^b^**	.03
**Major depression (reference category: no depression)**				
Anemia vs. no anemia	1.79 (1.02;3.15)	.04	1.75 (0.95;3.24) **^c^**	.07
**Minor depression (reference category: no depression)**				
Anemia vs. no anemia	1.79 (0.80;4.05)	.16	1.89 (0.83;4.29) **^d^**	.13

^a^ Model 1 unadjusted;

^b^ Model 2 adjusted for age, sex, smoking status, chronic conditions, and functional limitations;

^c^ Model 2 adjusted for age, sex, BMI, smoking status, chronic conditions, and functional limitations; and

^d^ Model 2 adjusted for sex, and income.

Table 3 shows the effect of anemia treatment on depression. In the fully adjusted models, participants who had untreated anemia had increased odds of depression (OR = 2.64, CI 1.31–5.33), major depression (OR = 2.48, CI 1.11–5.54), and minor depression (OR = 2.81, CI 1.06–7.44), when compared to nonanemic participants. However, the odds of depression, major depression, and minor depression were not statistically different among those with treated anemia compared to nonanemic participants (*p* > .05).

**TABLE 3:** Unadjusted and multivariable‐adjusted logistic regression models: associations between anemia and depression: the effect of anemia treatment on depression, baseline data: The ESA Services Study

	Model 1^a^		Model 2	
	OR (95% CI)	*p* value	OR (95% CI)	*p* Value
**Total depression (reference category: no depression)**				
Treated anemia vs. no anemia	1.41 (0.67; 2.96)	.91	1.22 (0.55; 2.68) ^b^	.62
Untreated anemia vs. no anemia	2.44 (1.29; 4.62)	.006	2.64 (1.31; 5.33) ^b^	.007
**Major depression (reference category: no depression)**				
Treated anemia vs. no anemia	1.55 (0.68; 3.52)	.29	1.24 (0.52; 2.98) ^c^	.63
Untreated anemia vs. no anemia	2.04 (0.97; 4.29)	.06	2.48 (1.11; 5.54) ^c^	.03
**Minor depression (reference category: no depression)**				
Treated anemia vs. no anemia	1.01 (0.24; 4.24)	.99	1.04 (0.24; 4.42) ^d^	.96
Untreated anemia vs. no anemia	2.63 (1.01; 6.88)	.05	2.81 (1.06; 7.44) ^d^	.04

^a^ Model 1 unadjusted;

^b^ Model 2 adjusted for age, sex, smoking status, chronic conditions, and functional limitations;

^c^ Model 2 adjusted for age, sex, BMI, smoking status, chronic conditions, and functional limitations; and

^d^ Model 2 adjusted for sex, and income.

Additional analyses considering the duration and frequency of symptoms of depression (major or minor) showed similar results in the fully adjusted multivariable model. There was an increase in the odds of depression (OR = 3.07, CI 1.22–7.74) in participants with untreated anemia as compared to those not reporting anemia and no difference in the odds of depression in those with treated anemia compared to those without anemia (OR = 1.25, CI 0.36–4.35). We also assessed the frequency of self‐reported anemia among those with depressive symptoms and whether or not they received antidepressants in the past 6 months. Of the 213 individuals with depression, 32.8% had used antidepressants, 60.6% were nonusers of antidepressants in the past 6 months, and 6.6% had no data on antidepressant use. The proportion of individuals using antidepressants and reporting anemia was 11.4%, while those not using antidepressants reporting anemia was 10.9%. There was no significant difference in the proportion of self‐reported anemia between antidepressant users and nonuserss (*p* = .894).

## 5 DISCUSSION

The current study contributes to present literature by being the first, to our knowledge, to report on the association between the presence of anemia and its treatment on depression in primary care community‐dwelling older adults aged 65 years and older covered by a public drug plan in the Quebec, Canada where residents are covered for their medical consultations.

In Canada, data on the iron status of older adults are scarce, with only a few studies that documented the prevalence of anemia in older adults aged 65 years and over. Prior data were collected in 1973 during the Nutrition Canada Survey and those collected in 2009 for the Canadian health measure survey with anemia estimates of 10.1% in women and 7.2% in men (Canada, 2012; Cooper et al., 2006). The prevalence of self‐reported anemia in the current sample was estimated to be 6.7%, consistent with previous data in older adults (Cooper et al., 2012). According to the WHO reports on the public health significance of anemia in different countries, the estimates of anemia prevalence in our Quebec sample suggest that anemia represents a mild public health problem when estimates range between 5.0% and 19.9% (WHO, 2011).

The current findings suggested that self‐reported anemia was associated with depression and untreated anemia was associated with close to a threefold increased odds of depression compared to participants with no anemia. Similar findings were observed for minor and major depression among participants with untreated anemia. In contrast, those with treated anemia did not have an increased odds of depression compared to those with no anemia after controlling for potential confounders.

The relationship between anemia and depression in old age has been explored in cross‐sectional (Corona et al., 2014; Onder et al., 2005; Stewart & Hirani, 2012) and longitudinal studies (Ahmed & Vasiliadis, 2021; Steptoe et al., 2012). The current findings are consistent with the results of earlier studies suggesting a link between anemia and depression in old age. In addition, an accumulating body of evidence indicates a plausible biological pathway between anemia and depression. Anemia contributes to altered brain neurotransmitter homeostasis through poor brain myelination and impaired monoamine metabolism. These alterations in the brain's homeostatic mechanisms can lead to emotional and psychological problems predisposing to depression diagnosis (Beard & Connor, 2003; Kim, 2014). Our findings did not show a significant difference between individuals without anemia and those treated for anemia in the odds of reporting depression, while there was a significant increase in the odds of depression among those with untreated anemia. These results are consistent and support findings from previous studies suggesting that improving brain oxygenation, particularly to frontotemporal and subcortical brain structures, which are involved in the depression pathway through the treatment of anemia, may improve brain function and reduce the risk of depression (Kusunoki et al., 1981; Li et al., 2018; Oda et al., 2003).

### 5.1 Strengths and limitations

There are a number of limitations that need to be considered when interpreting the results with respect to selection and information bias. First, comparisons were carried out between those included in the analysis, that is, those with complete RAMQ medication data (*n* = 1447) and those not included in the analysis but completed the questionnaire at baseline (*n* = 318). Participants not included in the current analyses were not different from those included with respect to age, sex, education, income, marital status, number of chronic conditions, alcohol consumption, and cigarette smoking (*p* > .05). However, those included were more likely obese than those not included (*p* = .03). Comparisons with respect to anemia and depression did not show any significant difference and therefore minimizing the risk of a selection bias. Second, anemia was self‐reported and may be subject to recall bias. Published research using self‐reported measures of anemia showed that close to 10% of participants had not declared that they were anemic, although they were after blood tests (Hisa et al., 2019). As we do not expect this bias to be differential between individuals with and without depression, the associations reported in the current study may be underestimates.

Third, we have not explored causes of anemia in our study, and the treatment was assessed by the presence of at least one oral or parenteral prescription for anemia treatment according to the Canadian guidelines. Our findings are only applicable to anemias responsive to pharmacologic therapy.

Fourth, the assessment of anemia and depression was done at the same time point. The cross‐sectional design does not allow to conclude the temporality of events and whether improvement in depression symptoms could also contribute to improvement of anemia. Finally, the results of the current study can be generalized to similar populations that include French‐speaking community‐living older adults, the majority‐white, who were covered under a public medical plan for their medical consultations and public drug plan for their medications. Future research should focus on replicating these findings in other, more diverse data sets using longitudinal data.

## 6 CONCLUSIONS AND IMPLICATIONS

Anemia was associated with higher odds of depression, and this association was similar in older adult men and women. The prevalence of depression was lowest among participants with treated anemia compared to those untreated. A higher odds of depression was also observed among participants with untreated anemia as compared to those without a history of anemia. Participants with treated anemia were not different from those with no history of anemia in their odds of depression after adjustment for potential confounders. Anemia is a treatable condition in old age (Weiss, 2015), and the biological pathway between anemia and depression should be further studied to elucidate the possible effect of screening and treatment of anemia in older adults. Future longitudinal studies should focus on better understanding the etiologic pathway between anemia and depression and confirm the role of treating anemia on symptoms of depression.

## AUTHOR CONTRIBUTIONS

T.A, C.L.L, D.B., and HM.V: study concept and design. T.A: statistical analysis. T.A: drafting of the manuscript. All authors: interpretation of data, preparation, and critical revision of the manuscript.

### PEER REVIEW

The peer review history for this article is available at https://publons.com/publon/10.1002/brb3.2973.

## Data Availability

For data requests, please contact Dr. Helen Maria Vasiliadis. Data sharing is subjected to rules governed by the ethics committee of the University of Sherbrooke.
